# Immune checkpoint inhibitor-related myocarditis during DUO-E regimen for endometrial cancer: The first case report in gynecologic oncology

**DOI:** 10.1016/j.gore.2025.101924

**Published:** 2025-08-21

**Authors:** Eri Yamabe, Hironori Yamamoto, Keita Asano, Taku Yasui, Masashi Fujita, Tsuyoshi Hisa, Miho Kitai

**Affiliations:** a Department of Gynecology, Osaka International Cancer Institute, 3-1-69 Otemae, Chuo-ku, Osaka 541-8567, Japan; b Department of Cardio-Oncology, Osaka International Cancer Institute, 3-1-69 Otemae, Chuo-ku, Osaka 541-8567, Japan

**Keywords:** Endometrial cancer, DUO-E regimen, Immune checkpoint inhibitor, Myocarditis, Immune-related adverse event, Durvalumab

## Abstract

**Background:**

Immune checkpoint inhibitors (ICIs) are increasingly used in gynecologic oncology, with the DUO-E regimen—carboplatin, paclitaxel, and durvalumab—recently approved in Japan for advanced or recurrent endometrial cancer. However, ICI-related myocarditis (ICI-M) is a rare but potentially fatal adverse event requiring prompt recognition and treatment.

**Case Presentation:**

A 74-year-old woman with advanced endometrial cancer developed ICI-M following four cycles of DUO-E therapy. Sixteen days post-treatment, she presented with fever and fatigue. Markedly elevated troponin I (39.8 ng/mL), new-onset complete right bundle branch block, and a reduced left ventricular ejection fraction (40 %) raised suspicion for ICI-M. Endomyocardial biopsy revealed diffuse CD8 + T-cell infiltration and myocyte necrosis, consistent with active myocarditis. High-dose methylprednisolone (1 g/day) was promptly initiated, resulting in rapid cardiac recovery and clinical stabilization. Chemotherapy was successfully resumed after steroid tapering.

**Conclusion:**

This is the first reported case of ICI-M during DUO-E therapy for endometrial cancer. It underscores the importance of early clinical suspicion, rapid multidisciplinary collaboration, and timely intervention in managing ICI-M, particularly in high-risk patients. Continuous vigilance is warranted throughout the course of ICI therapy.

## Highlights

• A woman in her 70s with endometrial cancer developed ICI-M after four cycles of DUO-E.
• High-dose methylprednisolone rapidly improved her cardiac function and clinical status.
• Chemotherapy was successfully resumed after tapering of steroids.
• This is the first report of ICI-M during DUO-E therapy for endometrial cancer.
• Continuous vigilance is warranted throughout the course of ICI therapy.

## 1 Introduction

Immune checkpoint inhibitors (ICIs) are integral to gynecologic oncology treatment. The DUO-E regimen (carboplatin, paclitaxel, and durvalumab) is a first-line therapy for advanced or recurrent endometrial cancer, approved for reimbursement in Japan in 2024.

However, ICIs can induce immune-related adverse events (irAEs), including the rare but potentially fatal ICI-related myocarditis (ICI-M), necessitating prompt recognition and treatment. We report a case of endometrial cancer complicated by ICI-M during DUO-E therapy, highlighting the clinical features that facilitated early diagnosis, management strategies employed, and the critical role of multidisciplinary collaboration.

## 2 Case report

A 74-year-old woman with hypothyroidism treated with levothyroxine developed lower abdominal pain, dysuria, and abnormal genital bleeding. Transvaginal ultrasonography revealed a 3.0- × 2.5-cm intrauterine mass, and endometrial biopsy confirmed adenocarcinoma. Imaging showed intrauterine and peritoneal masses with widespread dissemination. Diagnostic laparoscopy confirmed extensive peritoneal disease and obliteration of the pouch of Douglas. Because optimal debulking was not feasible, partial omental biopsy was performed. Histopathology revealed high-grade adenocarcinoma with features suggestive of serous carcinoma (PAX8+, ER+, p53 overexpression, WT-1−, and pMMR), although endometrioid and clear cell histologies could not be excluded. The patient was diagnosed with FIGO stage IVB endometrial cancer and started on DUO-E therapy: carboplatin (AUC 6), paclitaxel (175 mg/m^2^), and durvalumab (1120 mg/body). The first three cycles were uneventful with a partial CT response. The fourth cycle was complicated by Grade 2 neutropenia, which resolved with supportive care.

Sixteen days after the fourth cycle, the patient developed a high-grade fever (38 °C–39 °C) and fatigue. Although the fever temporarily subsided the following day, progressive dyspnea and anorexia developed, prompting emergency hospitalization on Day 80 of treatment (4 days after fever onset).

Vital signs upon admission were as follows: temperature, 36.6 °C; blood pressure, 94/59 mmHg; heart rate, 85 bpm; SpO_2_, 98 % on room air; and Glasgow Coma Scale score, E4V5M6. Laboratory testing showed a leukocyte count of 3370/μL and C-reactive protein of 2.87 mg/dL, indicating moderate inflammation. Serum enzyme levels were elevated: aspartate aminotransferase, 208 U/L; alanine aminotransferase, 61 U/L; creatine kinase (CK), 936 U/L; and lactate dehydrogenase (LDH), 658 U/L. Notably, troponin I was markedly elevated at 39.8 ng/mL. Thyroid function was normal.

Considering the fever and fatigue, an infectious etiology was initially suspected. Polymerase chain reaction assays for SARS-CoV-2 and influenza virus were negative. Because the fever spontaneously resolved without antibiotics, C-reactive protein was only modestly elevated, and the leukocyte count was low (3370/μL), sepsis was considered unlikely. Nevertheless, blood cultures were obtained and later confirmed negative.

**Fig. 1:**
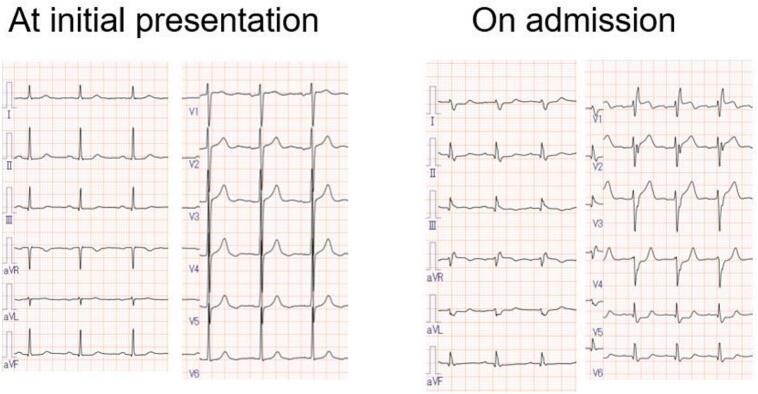
Electrocardiograms at initial outpatient evaluation and emergency admission.

The elevated hepatic transaminases raised concern for liver injury, including irAE hepatitis and viral and drug-induced causes. However, concurrent marked elevations in CK, LDH, and troponin I shifted suspicion toward myocardial injury, prompting urgent cardiac evaluation. Electrocardiography revealed new-onset complete right bundle branch block (CRBBB) (Fig. 1), and transthoracic echocardiography showed a reduced left ventricular ejection fraction (LVEF) of approximately 40 % (baseline: 63 %). Given the risk of life-threatening arrhythmias, a temporary transvenous pacing catheter was prophylactically inserted. Coronary angiography revealed no significant stenosis, ruling out ischemic heart disease. Given these findings and the ICI treatment history, ICI-M was suspected.

Given the clear evidence of significant myocardial injury, an endomyocardial biopsy of the right ventricular septum was promptly performed. Cardiac magnetic resonance imaging was not performed because the biopsy had already yielded definitive diagnostic information. Upon admission, serological testing was also performed for Epstein–Barr virus, along with assays for hepatitis B virus DNA, hepatitis C virus RNA, and antibodies to herpes simplex virus, varicella-zoster virus, and cytomegalovirus. All results were confirmed negative.

**Fig. 2:**
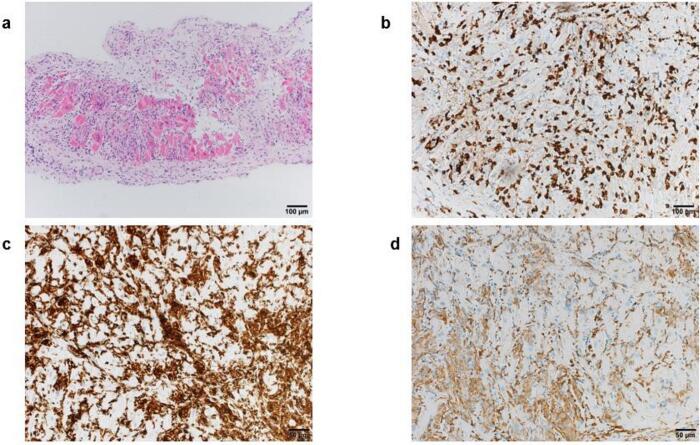
Histopathological results of endomyocardial biopsy. (a) Diffuse lymphocytic infiltration with extensive myocyte injury (hematoxylin and eosin staining). (b) Abundant CD8-positive T cells in the inflamed myocardium. (c) Presence of CD4-positive T cells in the same region as (b). (d) Numerous CD163-positive macrophages infiltrating the affected myocardial tissue.

Histopathological analysis revealed diffuse lymphocytic infiltration, predominantly CD8- and CD4-positive T cells, along with CD163-positive histiocytes. CD8-positive cells were infiltrating myocardial fibers, and widespread myocyte degeneration and necrosis were present, consistent with the Dallas criteria for active myocarditis (Fig. 2). These findings confirmed ICI-M.

**Fig. 3:**
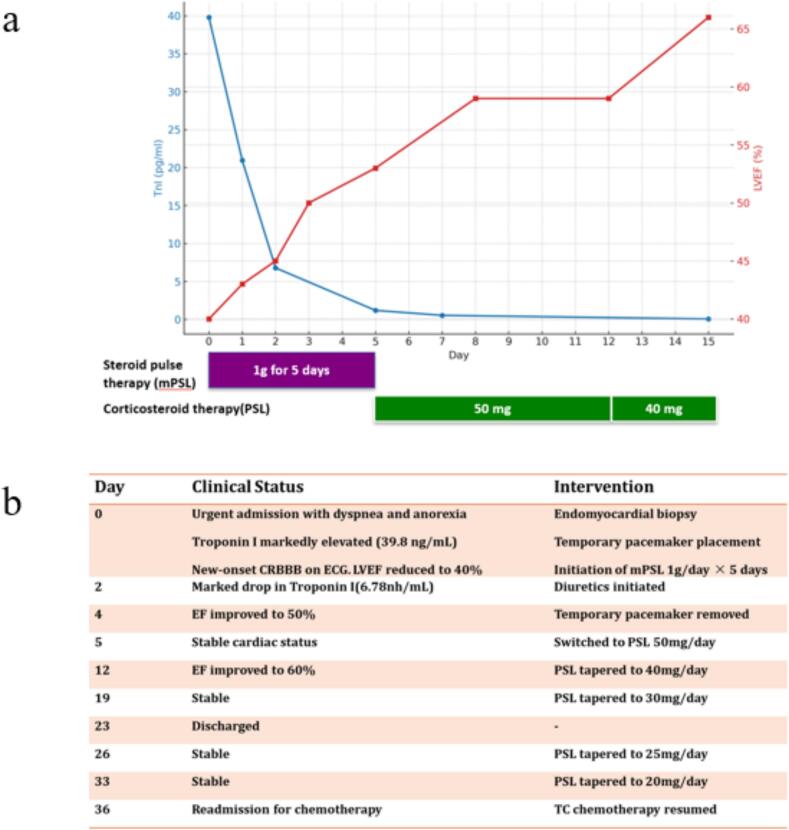
Clinical course. (a) Trends in LVEF and troponin I levels during corticosteroid therapy. (b) Clinical timeline.

We initiated high-dose methylprednisolone (1 g/day) on the day of diagnosis. By day 5, her LVEF had improved to >50 % with reduced arrhythmic risk, allowing for pacing catheter removal. We transitioned her to oral prednisolone 1 mg/kg/day. Troponin I and other enzyme levels decreased rapidly, and the LVEF recovered to nearly 60 % by day 16 (Fig. 3). Although CRBBB persisted, no arrhythmia occurred, and the patient’s overall condition stabilized.

Steroids were cautiously tapered by 5–10 mg/day every 7 days based on the clinical status. Chemotherapy was resumed once prednisolone reached 20 mg/day, and carboplatin and paclitaxel were reinitiated on day 36 post-myocarditis onset. The patient continued treatment with stable disease (Fig. 3).

## 3 Discussion

ICIs are widely used in gynecologic oncology, and DUO-E has emerged as a novel first-line treatment for advanced or recurrent endometrial cancer. The DUO-E trial demonstrated efficacy even in cases with pMMR, leading to its approval for reimbursement in Japan in 2024. ICIs are associated with various irAEs, including the rare but life-threatening ICI-M (incidence of 0.06–1.00 % and fatality rate of 25–50 %) (Mahmood et al., 2018, Wang et al., 2018).

In thoracic oncology, durvalumab-containing regimens have been linked to myocarditis with elevated troponin, impaired cardiac function, and conduction abnormalities, often resolving with early corticosteroid therapy (Nishikawa et al., 2023). Durvalumab-associated pericarditis and pericardial effusion have also been reported, suggesting cardiotoxicity beyond the myocardium (Kumar et al., 2017).

To our knowledge, this is the first report of ICI-M during DUO-E therapy for endometrial cancer in gynecologic oncology. Histopathology showed diffuse lymphocytic infiltration, predominantly CD8-positive T cells, with marked myocyte degeneration and necrosis—consistent with active myocarditis per the Dallas criteria. CD163-positive macrophage infiltration suggested a cooperative inflammatory response involving cytotoxic T cells and macrophages (Baldeviano et al., 2010, Johnson et al., 2016).

ICI-M onset is highly variable, and the clinical course is heterogeneous. Although most cases occur within 30 days of ICI initiation (median: 28–34 days) and 64 % develop after the first or second dose (Hu et al., 2019, Atallah-Yunes et al., 2021), no definitive timing criteria exist. Late-onset cases (up to 9 months after ICI discontinuation) have also been described, highlighting considerable clinical variability.

In this case, myocarditis developed on day 80, beyond the median but within the reported range. This reinforces that ICI-M can arise anytime during treatment and underscores the importance of continuous cardiac monitoring.

Some *meta*-analyses and pharmacovigilance data suggest a slightly higher myocarditis risk with PD-1 inhibitors (e.g., pembrolizumab, nivolumab) than with PD-L1 inhibitors (e.g., durvalumab, atezolizumab). However, considering the rarity of this adverse event and the data heterogeneity, this risk difference remains inconclusive. Severe, life-threatening myocarditis has been reported with both drug classes, necessitating vigilance regardless of ICI subtype (Salem et al., 2021).

Beyond ICI subtype, patient-related factors also influence ICI-M risk. A U.S. FDA Adverse Event Reporting System (FAERS) analysis showed that age of ≥75 years (odds ratio: 7.61) and female sex (odds ratio: 1.92) significantly increase ICI-M risk (Salem et al., 2019). Although our patient was female, she was 74 years old, marginally below the age threshold and thus warranting close surveillance. She also had hypothyroidism. Although autoimmune conditions are potential risk factors, their definitive association with ICI-M remains unproven (Mahmood et al., 2019). These considerations underscore the challenges of individualized risk stratification and the importance of vigilant monitoring during ICI therapy.

ICI-M is associated with poor prognostic factors, including markedly increased troponin I, neuromuscular complications, pre-existing cardiac disease, life-threatening arrhythmias or heart failure, and delayed therapy initiation (Mahmood et al., 2018, Tang et al., 2020, Zhang et al., 2021).

Risk factor analysis in this case, along with the clinical response and outcome, offers key insights. Despite markedly increased troponin I, the patient showed no neuromuscular complications, thereby avoiding one of the most severe prognostic indicators. She also had no history of cardiovascular disease and maintained normal cardiac function prior to ICI initiation, which may have supported cardiac stability during the acute phase.

Upon admission, new-onset CRBBB was identified, absent prior to treatment. Anticipating possible progression to complete atrioventricular block, temporary pacing was promptly initiated. Hemodynamic management, including diuretics, was also implemented to prevent worsening heart failure. These interventions were likely critical for avoiding life-threatening arrhythmia and circulatory collapse.

Among management factors, intervention timing was arguably the most critical to prognosis. When corticosteroids are initiated within 24 h of symptom onset, major adverse cardiovascular events occur in approximately 7.0 % of patients, versus 85.1 % with delays beyond 72 h (Tang et al., 2020).

Patients who respond rapidly to corticosteroids typically show improvement in cardiac function and biomarkers within 1 week (Mahmood et al., 2018, Zhang et al., 2021). By contrast, approximately 20–30 % require additional immunosuppressants due to insufficient response within 24–48 h. These refractory cases are often associated with delayed recognition and corticosteroid initiation, both linked to worse outcomes and increased need for second-line therapy (Salem et al., 2018; Zhang et al., 2021).

In this case, although initial symptoms were nonspecific (fever and fatigue), the marked troponin I elevation and electrocardiographic changes raised early suspicion of ICI-M. Prompt multidisciplinary intervention, including cardiologic evaluation, endomyocardial biopsy, and immediate initiation of high-dose methylprednisolone therapy (1 g/day), likely prevented deterioration and contributed to the favorable outcome. This case underscores the importance of early recognition, risk stratification, and timely intervention in managing ICI-M.

ICI-M frequently presents with nonspecific symptoms, making differential diagnosis challenging. When initial signs include low-grade fever and malaise, distinguishing ICI-M from viral myocarditis becomes a key clinical concern.

A definitive viral myocarditis diagnosis requires viral genome detection in blood or myocardial tissue, although positivity rates are often low in clinical practice (Kindermann et al., 2012). In this case, polymerase chain reaction assays for SARS-CoV-2 and influenza were negative, as were serologic tests for multiple viruses, including cytomegalovirus. Histopathology revealed diffuse CD8-positive T-cell infiltration and myocyte necrosis, findings characteristic of ICI-M.

Given the substantial clinical overlap between viral myocarditis and ICI-M, early and definitive differentiation is often difficult. In many cases, empirical treatment must begin alongside diagnostic evaluation. Although viral cultures were not performed in this case, the histologic findings of lymphocytic myocarditis, combined with rapid troponin I normalization and LVEF recovery upon corticosteroid monotherapy, strongly supported ICI-M. Establishing a faster, more precise diagnostic framework for patients with nonspecific symptoms is essential.

Notably, ICI-M often occurs as part of a severe multisystem inflammatory syndrome involving multiple organs. Early diagnosis and prompt treatment require coordinated, multidisciplinary efforts centered around the oncologist. Recently, the establishment of dedicated Immuno Checkpoint Inhibitor Proper (ICIP) use support teams has been recommended to facilitate comprehensive care of patients receiving ICIs (Salem et al., 2018).

In this case, early nonspecific symptoms (fever and fatigue) together with elevated troponin and electrocardiographic changes prompted cardiology consultation and led to ICI-M diagnosis. Collaboration with the ICIP team enabled preemptive planning for second-line immunosuppressants (infliximab, intravenous immunoglobulin, plasmapheresis, and abatacept) in case of steroid resistance. According to the 2020 NCCN guidelines, high-dose corticosteroids (e.g., methylprednisolone 1 g/day for 3–5 days) are recommended for Grade 3–4 ICI-M, followed by a gradual taper over 4–6 weeks. In this case, methylprednisolone was administered at 1 g/day for 5 days. Because clinical improvement occurred within 24 h, no additional immunosuppressive therapy was needed. The patient was transitioned to oral prednisolone with tapering based on clinical response, allowing successful chemotherapy resumption.

Given the potential for irAEs to interrupt or delay cancer therapy, early recognition and multidisciplinary collaboration are essential for maintaining treatment continuity and guiding clinical practice.

This report presents the first known case of ICI-M during DUO-E therapy for endometrial cancer in gynecologic oncology. The clinical course—in which early nonspecific symptoms prompted timely assessment and intervention, resulting in favorable cardiac recovery and successful chemotherapeutic reintroduction—offers practical implications for ICI-M management.

## 4 Conclusion

This case involves a woman with established risk factors for ICI-M who achieved a favorable outcome due to early clinical suspicion based on nonspecific symptoms and prompt initiation of appropriate therapy. Major cardiovascular events were thus avoided, and chemotherapy was successfully resumed.

Given the variable onset and presentation of ICI-M, sustained monitoring and early intervention are critical. This case provides clinically meaningful insights into ICI-M management in gynecologic oncology, highlighting the importance of early recognition and multidisciplinary collaboration in improving patient outcomes.

## Ethics approval and consent to participate and consent for publication

Written informed consent was obtained from the patient, and this case report was approved by the Institutional Review Board of Osaka International Cancer Institute according to the ethical standards laid down in the Declaration of Helsinki.

## CRediT authorship contribution statement

**Eri Yamabe:** Writing – original draft, Software, Resources, Project administration, Methodology, Investigation, Funding acquisition, Formal analysis, Data curation, Conceptualization. **Hironori Yamamoto:** Writing – review & editing. **Keita Asano:** Investigation. **Taku Yasui:** Investigation. **Masashi Fujita:** Investigation. **Tsuyoshi Hisa:** Investigation. **Miho Kitai:** Writing – review & editing, Investigation.

## Declaration of Competing Interest

The authors declare that they have no known competing financial interests or personal relationships that could have appeared to influence the work reported in this paper.
